# Cerebrospinal Fluid P-Tau_181P_: Biomarker for Improved Differential Dementia Diagnosis

**DOI:** 10.3389/fneur.2015.00138

**Published:** 2015-06-17

**Authors:** Hanne Struyfs, Ellis Niemantsverdriet, Joery Goossens, Erik Fransen, Jean-Jacques Martin, Peter P. De Deyn, Sebastiaan Engelborghs

**Affiliations:** ^1^Reference Center for Biological Markers of Dementia (BIODEM), Institute Born-Bunge, University of Antwerp, Antwerp, Belgium; ^2^StatUa Center for Statistics, University of Antwerp, Antwerp, Belgium; ^3^Biobank, Institute Born-Bunge, University of Antwerp, Antwerp, Belgium; ^4^Department of Neurology and Memory Clinic, Hospital Network Antwerp (ZNA) Middelheim and Hoge Beuken, Antwerp, Belgium; ^5^Department of Neurology and Alzheimer Research Center, University Medical Center Groningen (UMCG), Groningen, Netherlands

**Keywords:** Alzheimer’s disease, dementia, differential diagnosis, biomarkers, cerebrospinal fluid, neuropathology, tau

## Abstract

The goal of this study is to investigate the value of tau phosphorylated at threonine 181 (P-tau_181P_) in the Alzheimer’s disease (AD) cerebrospinal fluid (CSF) biomarker panel for differential dementia diagnosis in autopsy confirmed AD and non-AD patients. The study population consisted of 140 autopsy confirmed AD and 77 autopsy confirmed non-AD dementia patients. CSF concentrations of amyloid-β peptide of 42 amino acids (Aβ_1–42_), total tau protein (T-tau), and P-tau_181P_ were determined with single analyte ELISA-kits (INNOTEST^®^, Fujirebio, Ghent, Belgium). Diagnostic accuracy was assessed through receiver operating characteristic (ROC) curve analyses to obtain area under the curve (AUC) values and to define optimal cutoff values to discriminate AD from pooled and individual non-AD groups. ROC curve analyses were only performed on biomarkers and ratios that differed significantly between the groups. Pairwise comparison of AUC values was performed by means of DeLong tests. The Aβ_1–42_/P-tau_181P_ ratio (AUC = 0.770) performed significantly better than Aβ_1–42_ (AUC = 0.677, *P* = 0.004), T-tau (AUC = 0.592, *P* < 0.001), and Aβ_1–42_/T-tau (AUC = 0.678, *P* = 0.001), while P-tau_181P_ (AUC = 0.720) performed significantly better than T-tau (AUC = 0.592, *P* < 0.001) to discriminate between AD and the pooled non-AD group. When comparing AD and the individual non-AD diagnoses, Aβ_1–42_/P-tau_181P_ (AUC = 0.894) discriminated AD from frontotemporal dementia significantly better than Aβ_1–42_ (AUC = 0.776, *P* = 0.020) and T-tau (AUC = 0.746, *P* = 0.004), while P-tau_181P_/T-tau (AUC = 0.958) significantly improved the differentiation between AD and Creutzfeldt-Jakob disease as compared to Aβ_1–42_ (AUC = 0.688, *P* = 0.004), T-tau (AUC = 0.874, *P* = 0.040), and Aβ_1–42_/P-tau_181P_ (AUC = 0.760, *P* = 0.003). In conclusion, this study demonstrates P-tau_181P_ is an essential component of the AD CSF biomarker panel, and combined assessment of Aβ_1–42_, T-tau, and P-tau_181P_ renders, to present date, the highest diagnostic power to discriminate between AD and non-AD dementias.

## Introduction

The clinical diagnosis of Alzheimer’s disease (AD) is mainly based on the exclusion of other diseases (1). Relative to autopsy confirmation, the clinical diagnostic criteria of AD (1) reach on average 81% sensitivity and 70% specificity (2). However, these figures mostly originate from specialized clinical centers and from diagnoses based on follow-up periods of several years. In the earliest stages of the disease and when the diagnostic work-up is performed in non-specialized centers, far lower diagnostic accuracy can be expected. Diagnosis of definite AD can therefore only be made through postmortem pathological examination of the brain.

Analyzing cerebrospinal fluid (CSF) levels of amyloid-β peptide of 42 amino acids (Aβ_1–42_), total tau protein (T-tau) and tau phosphorylated at threonine 181 (P-tau_181P_) increases diagnostic certainty for AD (3). Based on autopsy confirmation, it was shown that in the majority of patients with a clinically ambiguous diagnosis (when the clinical diagnostic work-up was not able to discriminate between AD and a non-AD dementia), a correct diagnosis would have been established in 82% by using these CSF biomarkers, indicating that CSF biomarkers may have a particular added diagnostic value in patients with ambiguous clinical diagnoses (4).

Compared to controls, decreased Aβ_1–42_ and increased T-tau and/or P-tau_181P_ concentrations are found in AD. However, when compared to non-AD dementias, the differences are less obvious as the concentrations in patients with non-AD dementias are generally intermediate compared to those found between controls and AD patients, thus pointing to an overlap between AD and non-AD patients, especially in dementia with Lewy bodies (DLB) and to a lesser extent in frontotemporal dementia (FTD), vascular dementia (VaD), and Creutzfeldt-Jakob’s disease (CJD) (5). This overlap may partly be explained by the presence of mixed pathologies as well as the low sensitivity and specificity of the clinical diagnosis as most biomarker studies rely on clinically diagnosed patients.

The goal of this study is to investigate the value of P-tau_181P_ in the AD CSF biomarker panel for differential dementia diagnosis in autopsy confirmed AD and non-AD patients.

## Materials and Methods

### Study population

In brief, the study population consisted of 140 and 77 CSF samples from dementia patients with pathologically confirmed diagnoses of AD and non-AD, respectively. All CSF samples were selected from the Biobank, Institute Born-Bunge, Antwerp, Belgium. Samples from 173 dementia patients were collected in the Memory Clinic of the Hospital Network Antwerp (ZNA, Antwerp, Belgium) between January 1992 and May 2008, whereas samples from 44 dementia patients were collected in referring centers between April 1992 and May 2005.

The study was approved by the local ethics committee (CME Middelheim) and all subjects gave written informed consent.

### Pathological criteria

All pathological diagnoses were established according to standard neuropathological criteria by the same neuropathologist (Jean-Jacques Martin). Although the neuropathologist was blinded for the CSF biomarker data, he had access to all neuroimaging data and the clinical files of the patients included. For the diagnosis of AD, VaD (*n* = 18), and DLB (*n* = 24), the neuropathological criteria of Montine et al. (6) were applied. FTD (*n* = 17) was neuropathologically diagnosed according to the Cairns criteria (7) and Mackenzie criteria (8, 9). CJD (*n* = 13) was diagnosed according to the criteria of Markesbery (10). Mixed dementia (MXD) was diagnosed when the patient fulfilled the neuropathological criteria of AD in combination with minor pathology suggestive of cerebrovascular disease (*n* = 12), DLB (*n* = 1), or Parkinson’s disease (*n* = 1). For statistical analyses, the MXD group (*n* = 14) was pooled with the AD group. The pooled non-AD group furthermore consisted of few patients with progressive nuclear palsy (*n* = 3), spinocerebellar ataxia (*n* = 1), and normal pressure hydrocephalus combined with VaD (*n* = 1). Neuropathology was performed on the right hemisphere of the brain.

### CSF analyses

All subjects underwent a lumbar puncture (LP) in order to collect CSF. LP was performed between the intervertebral space L3/L4 or L4/L5 (11). CSF was sampled according to a standard protocol (12). All samples were stored in polypropylene vials to avoid adsorption of Aβ to the wall of the vial. The samples were frozen in liquid nitrogen and stored at −80°C until analysis.

CSF concentrations of Aβ_1–42_, T-tau, and P-tau_181P_ were determined with commercially available single analyte ELISA-kits (respectively, INNOTEST^®^ β-AMYLOID_(1–42)_, INNOTEST^®^ hTAU-Ag, and INNOTEST^®^ PHOSPHO-TAU_(181P)_; Fujirebio, Ghent, Belgium). A complete description of the CSF analysis has been published previously (13).

### Statistical analyses

Statistical analyses were performed using SPSS 20. As most variables were not normally distributed, non-parametric tests were used. To compare gender distribution between the groups, a Chi-square test was performed. Subsequently, Mann–Whitney *U* tests were performed to compare clinical and biomarker data between the groups. Receiver operating characteristic (ROC) curve analyses were used to obtain area under the curve (AUC) values and to define optimal cutoff values to discriminate AD from the pooled and individual non-AD groups. ROC curve analyses were only performed on biomarkers and ratios that were significantly different (*P* < 0.05), based on the Mann–Whitney *U* tests. The cutoff values were determined by calculating the maximal sum of sensitivity and specificity (i.e., maximizing the Youden index). In order to pairwise compare AUC values, DeLong tests were performed using the pROC package (14) in the statistical software package R (R Core Team).

### Systematic review

To be able to compare the results of this study, a systematic review on the diagnostic accuracy of P-tau_181P_ for differential dementia diagnosis was performed. A PubMed search (until May 2015) was performed using the following terms: (Cerebrospinal fluid OR CSF) AND diagnos* AND (Alzheimer* OR AD OR dementia) AND (tau OR beta amyloid OR abeta) AND (sensitivity OR specificity). Only publications in the English language were evaluated. Subsequently, relevant publications were searched for in reference lists. Publications were included when: (a) their aim was to improve the diagnostic accuracy of diagnosis of dementia by means of CSF biomarkers, (b) AD patients and pooled non-AD patients or patients with DLB, FTD, VaD and/or CJD were included, (c) P-tau_181P_ together with Aβ_1–42_ and/or T-tau was measured in CSF, and (d) diagnostic accuracy values were reported (AUC, sensitivity, and/or specificity). Publications comparing only AD to healthy control subjects were not considered.

## Results

**Figure 1:**
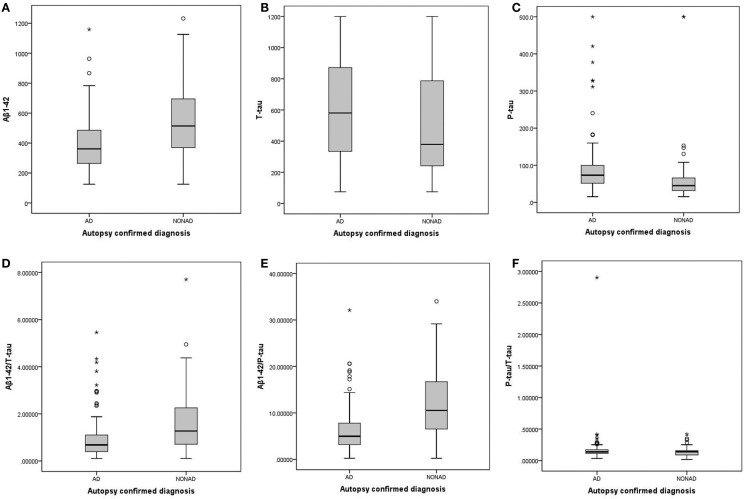
**Boxplots of the individual biomarkers and ratios, comparing AD and non-AD**. **(A)** Aβ_1–42_; **(B)** T-tau; **(C)** P-tau_181P_; **(D)** Aβ_1–42_/T-tau; **(E)** Aβ_1–42_/P-tau_181P_; **(F)** P-tau_181P_/T-tau. AD, Alzheimer’s disease; non-AD, dementia not due to Alzheimer’s disease; Aβ_1–42_, amyloid-β peptide of 42 amino acids; T-tau, total tau-protein; P-tau_181P_, tau phosphorylated at threonine 181.

Table 1 shows the demographic, clinical, and biomarker data of the studied population. The AD and non-AD groups were not age-matched. However, based on co-variate analyses, confounding effects of age on differences in biomarker concentrations were excluded. Therefore, no corrections for age were included in the subsequent analyses. Boxplots of the individual biomarkers and ratios are presented in Figure 1.

**Table 1:** **Demographic, clinical, and biomarker data of the study population**.

	AD	Non-AD	*P*-value
*N* (M/F)	140 (71/69)	77 (45/32)	0.275
Age at sampling (years)	76 (71–85)	72 (65–76)	**0.001**
MMSE (/30)	14 (9–19) (*n* = 98)	16 (9–21) (*n* = 51)	0.228
Years between sampling and death	0.0 (0.0–2.5)	0.0 (0.0–2.0)	0.452
Aβ_1–42_ (pg/mL)	361 (264–485)	514 (369–695)	**<0.001**
T-tau (pg/mL)	581 (335–872)	379 (242–787)	**0.025**
P-tau_181P_ (pg/mL)	73.2 (51.6–100.0)	45.0 (31.9–65.9)	**<0.001**
Aβ_1–42_/T-tau	0.682 (0.399–1.100)	1.273 (0.719–2.257)	**<0.001**
Aβ_1–42_/P-tau_181P_	4.982 (3.174–7.802)	10.535 (6.522–16.711)	**<0.001**
P-tau_181P_/T-tau	0.138 (0.113–0.171)	0.141 (0.090–0.158)	0.094

*All data are median values with 25th and 75th quartiles between brackets, except for *N*. To compare gender distribution between the groups, a Chi-square test was performed, while Mann–Whitney *U* tests were used to compare clinical and biomarker data between the groups*.

*AD, Alzheimer’s disease; non-AD, dementia not due to Alzheimer’s disease; MMSE, mini-mental state examination; Aβ_1–42_, amyloid-β peptide of 42 amino acids; T-tau, total tau-protein; P-tau_181P_, tau phosphorylated at threonine 181*.

*Bold values show statistically significant P-values (P < 0.05)*.

The diagnostic powers to discriminate between AD and non-AD of the individual biomarkers and ratios that were significantly different are shown in Table 2. Based on the DeLong tests (Table 3), the AUC of the Aβ_1–42_/P-tau_181P_ ratio was significantly different from those of Aβ_1–42_, T-tau, and Aβ_1–42_/T-tau, while the AUC of P-tau_181P_ differed significantly from the AUC of T-tau.

**Table 2:** **Diagnostic power of the significantly different individual biomarkers and ratios to discriminate between AD and non-AD, measured by ROC curve analyses**.

AD vs. non-AD	AUC	95% CI	Cutoff	Sens (%)	Spec (%)
Aβ_1–42_	0.677	0.597–0.757	500.27	79.3	53.2
T-tau	0.592	0.508–0.675	472.35	62.1	63.6
P-tau_181P_	0.720	0.648–0.792	50.35	77.9	61.0
Aβ_1–42_/T-tau	0.678	0.601–0.755	1.08	75.0	57.1
Aβ_1–42_/P-tau_181P_	0.770	0.703–0.837	9.11	82.9	59.7

*AD, Alzheimer’s disease; non-AD, dementia not due to Alzheimer’s disease; AUC, area under the curve; CI, confidence interval; sens, sensitivity; spec, specificity; Aβ_1–42_, amyloid-β peptide of 42 amino acids; T-tau, total tau-protein; P-tau_181P_, tau phosphorylated at threonine 181*.

**Table 3:** ***P* values of pairwise comparisons of AUC values of the ROC curve analyses to discriminate between AD and non-AD, using DeLong tests**.

	P-tau_181P_	A**β**_1–42_/P-tau_181P_
Aβ_1–42_	0.450	**0.004**
T-tau	**<0.001**	**<0.001**
Aβ_1–42_/T-tau	0.290	**0.001**
Aβ_1–42_/P-tau_181P_	0.100	NA

*Aβ_1–42_, amyloid-β peptide of 42 amino acids; T-tau, total tau-protein; P-tau_181P_, tau phosphorylated at threonine 181; NA, not applicable*.

*Bold values show statistically significant P-values (P < 0.05)*.

When comparing AD and the different non-AD diagnoses, the Aβ_1–42_/P-tau_181P_ ratio was significantly different in every differential diagnosis (Table 4). This also held true for P-tau_181P_, except for AD vs. CJD. On the other hand, P-tau_181P_/T-tau was found to be significantly different when comparing AD to CJD.

**Table 4:** ***P* values of pairwise comparisons of the individual biomarkers and ratios, measured by Mann–Whitney *U* tests**.

	AD vs. FTD	AD vs. DLB	AD vs. CJD	AD vs. VaD
Aβ_1–42_	**<0.001**	0.068	**0.025**	0.078
T-tau	**0.001**	0.051	**<0.001**	**0.019**
P-tau_181P_	**<0.001**	**0.011**	0.081	**0.001**
Aβ_1–42_/T-tau	**<0.001**	**0.008**	0.054	**0.010**
Aβ_1–42_/P-tau_181P_	**<0.001**	**0.002**	**0.002**	**0.003**
P-tau_181P_/T-tau	0.096	0.232	**<0.001**	0.932

*AD, Alzheimer’s disease; FTD, frontotemporal dementia; DLB, dementia with Lewy bodies; CJD, Creutzfeldt-Jakob disease; VaD, vascular dementia; Aβ_1–42_, amyloid-β peptide of 42 amino acids; T-tau, total tau-protein; P-tau_181P_, tau phosphorylated at threonine 181*.

*Bold values show statistically significant P-values (P < 0.05)*.

The diagnostic powers to discriminate between AD and the different non-AD diagnoses of the individual biomarkers and ratios that differed significantly are shown in Table 5. Based on the DeLong tests (Table 6), the Aβ_1–42_/P-tau_181P_ ratio performed significantly better than Aβ_1–42_ and T-tau to discriminate AD from FTD, while the AUC of P-tau_181P_/T-tau was significantly better than those of Aβ_1–42_, T-tau, and Aβ_1–42_/P-tau_181P_ to differentiate between AD and CJD.

**Table 5:** **Diagnostic power of the significantly different individual biomarkers and ratios to discriminate between AD and individual non-AD diagnoses, measured by ROC curve analyses**.

	AUC	95% CI	Cutoff	Sens (%)	Spec (%)
**AD vs. FTD**
Aβ_1–42_	0.776	0.652–0.900	385.31	57.1	88.2
T-tau	0.746	0.654–0.838	423.00	67.9	82.4
P-tau_181P_	0.810	0.710–0.910	47.25	81.4	76.5
Aβ_1–42_/T-tau	0.863	0.794–0.931	0.97	70.1	94.1
Aβ_1–42_/P-tau_181P_	0.894	0.823–0.965	9.77	86.4	82.4
**AD vs. DLB**
P-tau_181P_	0.664	0.539–0.788	59.05	65.7	70.8
Aβ_1–42_/T-tau	0.670	0.539–0.802	0.80	60.7	75.0
Aβ_1–42_/P-tau_181P_	0.694	0.565–0.824	8.46	80.0	58.3
**AD vs. CJD**
Aβ_1–42_	0.688	0.521–0.855	440.12	66.4	69.2
T-tau	0.874	0.775–0.973	>1200	84.3	92.3
Aβ_1–42_/P-tau_181P_	0.760	0.634–0.886	6.84	67.9	84.6
P-tau_181P_/T-tau	0.958	0.925–0.991	0.1030	84.3	100.0
**AD vs. VaD**
T-tau	0.670	0.534–0.807	467.93	62.1	72.2
P-tau_181P_	0.733	0.599–0.867	49.85	78.6	66.7
Aβ_1–42_/T-tau	0.687	0.569–0.804	0.72	56.4	77.8
Aβ_1–42_/P-tau_181P_	0.718	0.598–0.838	5.30	55.7	77.8

*AD, Alzheimer’s disease; FTD, frontotemporal dementia; DLB, dementia with Lewy bodies; CJD, Creutzfeldt-Jakob disease; VaD, vascular dementia; AUC, area under the curve; CI, confidence interval; sens, sensitivity; spec, specificity; Aβ_1–42_, amyloid-β peptide of 42 amino acids; T-tau, total tau-protein; P-tau_181P_, tau phosphorylated at threonine 181*.

**Table 6:** ***P* values of pairwise comparisons of AUC values of the ROC curve analyses to discriminate between AD and individual non-AD diagnoses, using DeLong tests**.

	P-tau_181P_	A**β**_1–42_/P-tau_181P_	P-tau_181P_/T-tau
**AD vs. FTD**
Aβ_1–42_	0.700	**0.020**	NA
T-tau	0.120	**0.004**	NA
Aβ_1–42_/T-tau	0.280	0.280	NA
**AD vs. DLB**
Aβ_1–42_/T-tau	0.890	0.360	NA
**AD vs. CJD**
Aβ_1–42_	NA	0.327	**0.004**
T-tau	NA	0.220	**0.040**
Aβ_1–42_/P-tau_181P_	NA	NA	**0.003**
**AD vs. VaD**
T-tau	0.370	0.600	NA
Aβ_1–42_/T-tau	0.600	0.610	NA

*AD, Alzheimer’s disease; FTD, frontotemporal dementia; DLB, dementia with Lewy bodies; CJD, Creutzfeldt-Jakob disease; VaD, vascular dementia; Aβ_1–42_, amyloid-β peptide of 42 amino acids; T-tau, total tau-protein; P-tau_181P_, tau phosphorylated at threonine 181; NA, not applicable*.

*Bold values show statistically significant P-values (P < 0.05)*.

The results of the systematic review are summarized in Table S1 in Supplementary Material. Only results comparing AD to non-AD, FTD, DLB, CJD, and/or VaD were included in this table.

## Discussion

The goal of this study was to investigate the value of P-tau_181P_ in the AD biomarker panel for differential dementia diagnosis. First of all, the ratio of Aβ_1–42_/P-tau_181P_ was shown to have a significantly higher diagnostic power than Aβ_1–42_, T-tau, and the Aβ_1–42_/T-tau ratio, while P-tau_181P_ was found to perform significantly better than T-tau to discriminate between AD and non-AD dementia. This clearly signifies the importance of P-tau_181P_ in the biomarker panel for differential dementia diagnosis. Our results are in line with previously reported findings of (combinations with) P-tau_181P_ having most power to discriminate between AD and non-AD dementias (12, 15–29).

However, in contrast to former studies performed in clinically diagnosed AD and pooled non-AD dementia patients (15, 21, 22, 26–28), the AUC, sensitivity, and specificity of neither P-tau_181P_ nor Aβ_1–42_/P-tau_181P_ reached the minimal level of 0.80, as established by the Consensus Report of the Working Group on Molecular and Biochemical Markers of AD (30). This is probably not due to the accuracy of the diagnoses used in this study, as autopsy confirmation was used. A possible explanation of the discrepancy in accuracy levels between this study and former studies could be the composition of the non-AD groups. As shown in this study, the accuracy levels of, for example, AD vs. FTD are substantially higher than those of AD vs. DLB. Therefore, if a non-AD group is primarily composed of FTD patients, the AUC levels may be higher than when DLB patients prevail in the non-AD group.

When focusing on the discrimination between AD and FTD, our results showed that the diagnostic power of Aβ_1–42_/P-tau_181P_ was significantly higher than those of Aβ_1–42_ and T-tau. These results confirm earlier studies performed in clinically diagnosed AD and FTD patients (16, 17, 24, 25, 29).

With regard to the differentiation between AD and CJD, the diagnostic power of P-tau_181P_/T-tau was significantly higher than those of Aβ_1–42_, T-tau, and Aβ_1–42_/P-tau_181P_. Our results confirm those of former studies performed in clinically diagnosed AD and CJD patients, and partly performed in autopsy confirmed cases (31–34).

In these latter two comparisons with individual non-AD groups, the AUCs did reach the minimal level of 0.80. This indicates that the pathophysiological variability in the pooled non-AD group lowers the diagnostic accuracy of the CSF biomarkers.

It should be noted that the ratios and other combinations of the AD CSF biomarkers should be used with care. Due to (pre-)analytical issues (35), concentrations differ exceedingly between laboratories. External quality controls and reference material might be able to reduce this variability, which would enable the general use of the same cutoff that was validated in a multicenter setting. At this moment, cutoffs for individual biomarkers as well as ratios and other combinations should be validated in-house before they can be used in clinical practice (36, 37).

In order to further increase diagnostic accuracy, other biomarkers should be included in the biomarker panel in the future. Examples of possible fluid biomarkers for features of Aβ processing in AD are β-site APP cleaving enzyme-1 (BACE1) activity (38–44), soluble amyloid precursor protein (sAPP) α and β (42, 44–51), and Aβ oligomers (52–60). Some fluid biomarkers that are still being investigated seem more specific for non-AD dementias and could also increase diagnostic accuracy when added to the biomarker panel. Examples of possible such non-AD biomarkers are TAR DNA-binding protein 43 (TDP-43) (61–63), TPD-43 phosphorylated at S409 (pTDP-43) (63), and progranulin (64–66) for FTD, α-synuclein (67–71) and neurosin (72) for DLB, metalloproteinases-9 for VaD (73, 74), and total CSF prion protein for CJD (75). For reviews on these biomarkers, see Ref. (76–80). Most of these biomarkers need extensive validation as well as validated ready-to-use analytical methods before they can be used in combination with Aβ_1–42_, T-tau, and P-tau_181P_ for differential dementia diagnosis in clinical practice.

Another highly promising approach is combining fluid biomarkers and imaging, such as magnetic resonance imaging (MRI) and positron emission tomography (PET) imaging. Several studies have shown that combinations of fluid and imaging biomarkers render higher diagnostic power than these modalities alone (81–85).

In conclusion, this study demonstrates P-tau_181P_ is a fundamental component of the AD biomarker panel and the combined assessment of Aβ_1–42_, T-tau, and P-tau_181P_ renders, to present date, the highest diagnostic power to discriminate between AD and non-AD dementias. New biomarkers more specifically targeted at non-AD dementia pathology should further increase diagnostic power in the future.

## Conflict of Interest Statement

The authors declare that the research was conducted in the absence of any commercial or financial relationships that could be construed as a potential conflict of interest.

## Supplementary Material

The Supplementary Material for this article can be found online at http://journal.frontiersin.org/article/10.3389/fneur.2015.00138
