# Supplementary material for: Cerebrospinal Fluid P-Tau181P: Biomarker for Improved Differential Dementia Diagnosis
Source: Front Neurol. 2015 Jun 17;6:138. doi: 10.3389/fneur.2015.00138 (PMC4470274; doi:10.3389/fneur.2015.00138)
Supplement: Supplementary file 1 [file table_1.docx]

***Supplementary Material***

**Cerebrospinal fluid P-tau_181P_: biomarker for improved differential dementia diagnosis**

**Hanne Struyfs^1^, Ellis Niemantsverdriet^1^, Joery Goossens^1^, Erik Fransen^2^, Jean-Jacques Martin^3^, Peter P. De Deyn^1,3,4,5^, Sebastiaan Engelborghs^1,4*^**

^1^Reference Center for Biological Markers of Dementia (BIODEM), Institute Born-Bunge, University of Antwerp, Antwerp, Belgium

^2^StatUa Center for Statistics, University of Antwerp, 2610 Antwerp, Belgium

^3^Biobank, Institute Born-Bunge, University of Antwerp, Antwerp, Belgium

^4^Department of Neurology and Memory Clinic, Hospital Network Antwerp (ZNA) Middelheim and Hoge Beuken, Antwerp, Belgium

^5^Department of Neurology and Alzheimer Research Center, University Medical Center Groningen (UMCG), Groningen, The Netherlands

*** Correspondence:** Prof. Dr. S. Engelborghs, Reference Center for Biological Markers of Dementia (BIODEM), University of Antwerp, Universiteitsplein 1, 2610 Antwerp, Belgium

[sebastiaan.engelborghs@uantwerpen.be](mailto:sebastiaan.engelborghs@uantwerpen.be)

# Supplementary Table

Supplementary Table 1. Diagnostic accuracy of the individual biomarkers and combinations of biomarkers in previously published studies. AD = Alzheimer’s disease; Aβ_1-42_ = amyloid-β peptide of 42 amino acids; AUC = area under the curve; CJD = Creutzfeldt-Jakob disease; DLB = dementia with Lewy bodies; FTD = frontotemporal dementia; non-AD = dementia not due to Alzheimer’s disease; NR = not reported; P-tau_181P_ = tau phosphorylated at threonine 181; sens = sensitivity; spec = specificity; T-tau = total tau-protein; VaD = vascular dementia.

| **Study** | **Groups** | **N** | **CSF biomarkers** | **AUC** | **cutoff** | **sens**  **(%)** | **spec**  **(%)** |
| --- | --- | --- | --- | --- | --- | --- | --- |
| **AD vs. non-AD** | | | | | | | |
| [**Maddalena et al. (2003**](#_ENREF_14)**)** | AD | 51 | Aβ_1-42_ | 0.731 | 0.49 ng/mL | 78.0 | 70.0 |
|  | non-AD | 30 | P-tau_181P_ | 0.710 | 35 pg/mL | 73.0 | 63.0 |
|  |  |  | P-tau_181P_/Aβ_1-42_ | 0.801 | 83 pg/mL | 80.0 | 73.0 |
| [**Olsson et al. (2005**](#_ENREF_17)**)^*^** | AD | 78 | Aβ_1-42_ | *NR* | 515 pg/mL | 91.0 | 75.0 |
|  | non-AD | 128 | T-tau | *NR* | 436 pg/mL | 83.0 | 89.0 |
|  |  |  | P-tau_181P_ | *NR* | 87.3 pg/mL | 72.0 | 95.0 |
| [**Lewczuk et al. (2008**](#_ENREF_13)**) ^*^** | AD | 53 | Aβ_1-42_ | *NR* | 197.5 pg/mL | 75.5 | 60.0 |
|  | non-AD | 15 | T-tau | *NR* | 86 pg/mL | 66.0 | 80.0 |
|  |  |  | P-tau_181P_ | *NR* | 47.9 pg/mL | 77.4 | 73.3 |
| [**Engelborghs et al. (2008**](#_ENREF_7)**)** | Definite AD | 51 | Aβ_1-42_ | *NR* | *NR* | *NR* | *NR* |
|  | Definite non-AD | 15 | T-tau | *NR* | *NR* | *NR* | *NR* |
|  |  |  | P-tau_181P_ | *NR* | *NR* | *NR* | *NR* |
|  |  |  | Model Aβ_1-42_ + P-tau_181P_ | 0.941 | 2.03 | 80.0 | 93.0 |
| [**Welge et al. (2009**](#_ENREF_25)**)** | AD | 44 | Aβ_1-42_ | 0.774 | 0.458 ng/mL | 86.0 | 64.0 |
|  | non-AD | 87 | T-tau | 0.711 | 0.266 ng/mL | 87.0 | 48.0 |
|  |  |  | P-tau_181P_ | 0.874 | 0.065 ng/mL | 87.0 | 83.0 |
|  |  |  | Aβ_1-42_/T-tau | 0.773 | 1.451 | 92.0 | 60.0 |
|  |  |  | Aβ_1-42_/P-tau_181P_ | 0.894 | 8.157 | 95.0 | 75.0 |
| [**Yakushev et al. (2010**](#_ENREF_26)**)** | AD | 24 | T-tau | 0.730 | 440 pg/mL | 54.0 | 92.0 |
|  | non-AD | 13 | P-tau_181P_ | 0.910 | 65 pg/mL | 71.0 | 100 |
| [**Gabelle et al. (2013**](#_ENREF_9)**)** | AD | 272 | Aβ_1-42_ | 0.760 | 519 pg/mL | 81.6 | 71.4 |
|  | non-AD | 370 | T-tau | 0.840 | 362 pg/mL | 81.2 | 78.4 |
|  |  |  | P-tau_181P_ | 0.870 | 61 pg/mL | 76.8 | 88.4 |
|  |  |  | Aβ_1-42_/T-tau | 0.860 | 2.48 | 86.7 | 78.7 |
|  |  |  | Aβ_1-42_/P-tau_181P_ | 0.880 | 15.1 | 85.2 | 84.0 |
|  |  |  | Model Aβ_1-42_ + P-tau_181P_ | 0.900 | -0.37 | 84.5 | 86.5 |
| [**Shea et al. (2013**](#_ENREF_22)**)** | AD | 24 | Aβ_1-42_ | 0.690 | 301.6 pg/mL | 63.0 | 83.0 |
|  | non-AD | 12 | T-tau | 0.670 | 370.2 pg/mL | 83.0 | 58.0 |
|  |  |  | P-tau_181P_ | 0.740 | 31.51 pg/mL | 100 | 42.0 |
|  |  |  | Aβ_1-42_/T-tau | 0.740 | 1.54 | 96.0 | 50.0 |
|  |  |  | Aβ_1-42_/P-tau_181P_ | 0.800 | 6.87 | 79.0 | 75.0 |
| [**Duits et al. (2014**](#_ENREF_6)**)** | AD | 631 | Aβ_1-42_ | 0.800 | 550 pg/mL | 82.0^#^ | 72.0 |
|  | non-AD | 267 | T-tau | 0.790 | 375 pg/mL | 82.0^#^ | 63.0 |
|  |  |  | P-tau_181P_ | 0.810 | 52 pg/mL | 86.0^#^ | 59.0 |
|  |  |  | Aβ_1-42_ + T-tau and/or P-tau_181P_ abnormal |  |  | 74.0^#^ | 81.0 |
|  |  |  | ≥ 2 of 3 biomarkers abnormal |  |  | 86.0^#^ | 65.0 |
|  |  |  | T-tau/Aβ_1-42_ | 0.850 | 0.71 | 85.0^#^ | 75.0 |
|  |  |  | P-tau_181P_/Aβ_1-42_ | 0.860 | 0.11 | 85.0^#^ | 80.0 |
|  |  |  | Hulstaert model ([1999](#_ENREF_11)) (Aβ_1-42_ + T-tau) | 0.850 | 1 | 93.0^#^ | 65.0 |
|  |  |  | Mulder model ([2010](#_ENREF_16)) (Aβ_1-42_ + T-tau) | 0.850 | 1 | 93.0^#^ | 63.0 |
|  |  |  | Mattsson model ([2009](#_ENREF_15)) (Aβ_1-42_ + T-tau + P-tau_181P_) | 0.860 | 1 | 80.0^#^ | 80.0 |
|  |  |  | Schoonenboom model ([2012](#_ENREF_20)) (Aβ_1-42_ + P-tau_181P_) | 0.860 | 1 | 91.0^#^ | 72.0 |
| [**Seeburger et al. (2015**](#_ENREF_21)**)** | Definite AD | 92 | Aβ_1-42_ | *NR* | 463 pg/mL | 84.0 | 100 |
|  | Definite non-AD | 16 | T-tau | *NR* | 438 pg/mL | 73.0 | 94.0 |
|  |  |  | P-tau_181P_ | *NR* | 44 pg/mL | 90.0 | 60.0 |
|  |  |  | T-tau/Aβ_1-42_ | *NR* | 0.798 | 92.0 | 100 |
|  |  |  | P-tau_181P_/Aβ_1-42_ | *NR* | 0.131 | 88.0 | 100 |
| **AD vs. FTD** | | | | | | | |
| [**Schoonenboom et al. (2004**](#_ENREF_19)**)** | AD | 47 | Aβ_1-42_ | 0.860 | 413 pg/mL | 85.0 | 75.0 |
|  | FTD | 28 | T-tau | 0.813 | 377 pg/mL | 85.0 | 74.0 |
|  |  |  | P-tau_181P_ | 0.866 | 54 pg/mL | 85.0 | 82.0 |
| [**Blasko et al. (2006**](#_ENREF_3)**)** | AD | 23 | Aβ_1-42_ | *NR* | *NR* | *NR* | *NR* |
|  | FTD | 5 | T-tau | *NR* | *NR* | *NR* | *NR* |
|  |  |  | P-tau_181P_ | *NR* | *NR* | *NR* | *NR* |
|  |  |  | P-tau_181P_/Aβ_1-42_ | 0.900 | *NR* | 86.0 | 80.0 |
| [**Gabelle et al. (2011**](#_ENREF_10)**)** | AD | 52 | Aβ_1-42_ | 0.750 | 464 pg/mL | 79.0 | 62.0 |
|  | FTD | 34 | T-tau | 0.880 | 448 pg/mL | 88.0 | 82.0 |
|  |  |  | P-tau_181P_ | 0.950 | 58 pg/mL | 91.0 | 88.0 |
|  |  |  | Hulstaert model ([1999](#_ENREF_11)) (Aβ_1-42_ + T-tau) | 0.870 | 0.66 | 88.0 | 86.0 |
| [**de Souza et al. (2011**](#_ENREF_5)**)** | AD | 60 | Aβ_1-42_ | 0.817 | 292.1 pg/mL | 68.3 | 85.2 |
|  | FTD | 27 | T-tau | 0.832 | 458 pg/mL | 70.0 | 88.9 |
|  |  |  | P-tau_181P_ | 0.851 | 62.5 pg/mL | 83.3 | 85.2 |
|  |  |  | T-tau/Aβ_1-42_ | 0.926 | 1.23 | 95.0 | 85.2 |
|  |  |  | P-tau_181P_/Aβ_1-42_ | 0.942 | 0.211 | 91.7 | 92.6 |
| [**Irwin et al. (2013**](#_ENREF_12)**) ^*^** | Definite AD | 30 | Aβ_1-42_ | 0.874 | *NR* | *NR* | *NR* |
|  | Definite FTD | 10 | T-tau | 0.941 | *NR* | *NR* | *NR* |
|  |  |  | P-tau_181P_ | 0.889 | *NR* | *NR* | *NR* |
|  |  |  | T-tau/Aβ_1-42_ | 0.989 | 0.34 | *NR* | *NR* |
|  |  |  | P-tau_181P_/Aβ_1-42_ | 0.956 | *NR* | *NR* | *NR* |
| [**Ewers et al. (2015**](#_ENREF_8)**) ^*^** | AD | 167 | Aβ_1-42_ | *NR* | *NR* | 85.0 | 77.0 |
|  | FTD | 39 | T-tau | *NR* | *NR* | *NR* | *NR* |
|  |  |  | P-tau_181P_ | *NR* | *NR* | *NR* | *NR* |
|  |  |  | Model Aβ_1-42_ + P-tau_181P_ | *NR* | *NR* | 85.0 | 85.0 |
| **AD vs. DLB** | | | | | | | |
| [**Vanderstichele et al. (2006**](#_ENREF_23)**)** | AD | 94 | Aβ_1-42_ | *NR* | *NR* | *NR* | *NR* |
|  | DLB | 60 | T-tau | *NR* | *NR* | *NR* | *NR* |
|  |  |  | P-tau_181P_ ^¶^ | *NR* | 61 pg/mL | 80.0 | 79.0 |
| [**Wada-Isoe et al. (2007**](#_ENREF_24)**)** | AD | 24 | Aβ_1-42_ | *NR* | *NR* | *NR* | *NR* |
|  | DLB | 22 | P-tau_181P_ | *NR* | 46.3 pg/mL | 68.2 | 82.4 |
|  |  |  | P-tau_181P_/Aβ_1-42_ | *NR* | 13.3 | 72.7 | 70.6 |
| [**Aerts et al. (2011**](#_ENREF_1)**)** | AD | 44 | Aβ_1-42_ | 0.650 | 482 pg/mL | 62.0 | 65.0 |
|  | DLB | 21^†^ | T-tau | 0.950 | 294 pg/mL | 90.4 | 90.0 |
|  |  |  | P-tau_181P_ | 0.920 | 67 pg/mL | 81.0 | 95.0 |
|  |  |  | Model Aβ_1-42_ + T-tau + P-tau_181P_ | 0.960 | 0.42 | 92.9 | 90.0 |
| [**Ewers et al. (2015**](#_ENREF_8)**) ^*^** | AD | 167 | Aβ_1-42_ | *NR* | *NR* | 85.0 | 42.0 |
|  | DLB | 26 | T-tau | *NR* | *NR* | *NR* | *NR* |
|  |  |  | P-tau_181P_ | *NR* | *NR* | *NR* | *NR* |
|  |  |  | Model Aβ_1-42_ + P-tau_181P_ | *NR* | *NR* | 85.0 | 77.0 |
| **AD vs. FTD** | | | | | | | |
| [**Bahl et al. (2009**](#_ENREF_2)**)** | AD | 49 | T-tau | *NR* | 500 pg/mL | 67.0 | 100 |
|  | CJD | 21 | P-tau_181P_ | *NR* | *NR* | *NR* | *NR* |
|  |  |  | P-tau_181P_/T-tau | *NR* | 0.040 | 86.0 | 98.0 |
| **AD vs. VaD** | | | | | | | |
| [**de Jong et al. (2006**](#_ENREF_4)**)** | AD | 61^‡^ | Aβ_1-42_ | *NR* | 520 pg/mL | 82.0 | 76.0 |
|  | VaD | 25^‡^ | T-tau | *NR* | 321 pg/mL | 80.0 | 76.0 |
|  |  |  | P-tau_181P_ | *NR* | 68.5 pg/mL | 75.0 | 95.0 |
|  |  |  | T-tau/Aβ_1-42_ | *NR* | 1.2 | 82.0 | 92.0 |
|  |  |  | P-tau_181P_/Aβ_1-42_ | *NR* | 10.95 | 95.0 | 90.0 |
| [**Reijn et al. (2007**](#_ENREF_18)**)** | AD | 69 | Aβ_1-42_ | 0.730 | 540 ng/L | 87.0 | 62.0 |
|  | VaD | 26 | T-tau | 0.810 | 350 ng/L | 88.0 | 73.0 |
|  |  |  | P-tau_181P_ | 0.940 | 75 ng/L | 78.0 | 96.0 |
|  |  |  | Aβ_1-42_/P-tau_181P_ | 0.940 | 6.8 | 86.0 | 96.0 |
|  |  |  | Model Aβ_1-42_ + P-tau_181P_ | 0.960 | -1.1 | 91.0 | 96.0 |
|  |  |  | Aβ_1-42_**^*^** | 0.840 | 213 ng/L | 80.0 | 81.0 |
|  |  |  | T-tau**^*^** | 0.810 | 66 ng/L | 81.0 | 85.0 |
|  |  |  | P-tau_181P_**^*^** | 0.900 | 51 ng/L | 83.0 | 89.0 |
|  |  |  | Aβ_1-42_/P-tau_181P_**^*^** | 0.930 | 5.7 | 91.0 | 85.0 |
|  |  |  | Model Aβ_1-42_ + P-tau_181P_**^*^** | 0.930 | 0.11 | 96.0 | 85.0 |
| [**Ewers et al. (2015**](#_ENREF_8)**) ^*^** | AD | 167 | Aβ_1-42_ | *NR* | *NR* | 85.0 | 46.0 |
|  | VaD | 69 | T-tau | *NR* | *NR* | *NR* | *NR* |
|  |  |  | P-tau_181P_ | *NR* | *NR* | *NR* | *NR* |
|  |  |  | Model Aβ_1-42_ + P-tau_181P_ | *NR* | *NR* | 85.0 | 59.0 |
| **^*^** Biomarker levels determined with xMAP® technology (INNO-BIA AlzBio3, Innogenetics, Ghent).  ^#^ Sensitivity values are derived from the ROC curve analysis of AD vs. controls.  ^¶^ The classification tree retained only P-tau_181P_.  ^†^ DLB N = 20 for T-tau.  ^‡^ AD N = 56 and VaD N = 20 for P-tau_181P_. | | | | | | | |

# References

Aerts, M.B., Esselink, R.A., Claassen, J.A., Abdo, W.F., Bloem, B.R., and Verbeek, M.M. (2011). CSF tau, Abeta42, and MHPG differentiate dementia with Lewy bodies from Alzheimer's disease. *J Alzheimers Dis* 27**,** 377-384. doi: 10.3233/JAD-2011-110482.

Bahl, J.M., Heegaard, N.H., Falkenhorst, G., Laursen, H., Hogenhaven, H., Molbak, K., Jespersgaard, C., Hougs, L., Waldemar, G., Johannsen, P., and Christiansen, M. (2009). The diagnostic efficiency of biomarkers in sporadic Creutzfeldt-Jakob disease compared to Alzheimer's disease. *Neurobiol Aging* 30**,** 1834-1841. doi: 10.1016/j.neurobiolaging.2008.01.013.

Blasko, I., Lederer, W., Oberbauer, H., Walch, T., Kemmler, G., Hinterhuber, H., Marksteiner, J., and Humpel, C. (2006). Measurement of thirteen biological markers in CSF of patients with Alzheimer's disease and other dementias. *Dement Geriatr Cogn Disord* 21**,** 9-15. doi: 10.1159/000089137.

De Jong, D., Jansen, R.W., Kremer, B.P., and Verbeek, M.M. (2006). Cerebrospinal fluid amyloid beta42/phosphorylated tau ratio discriminates between Alzheimer's disease and vascular dementia. *J Gerontol A Biol Sci Med Sci* 61**,** 755-758.

De Souza, L.C., Lamari, F., Belliard, S., Jardel, C., Houillier, C., De Paz, R., Dubois, B., and Sarazin, M. (2011). Cerebrospinal fluid biomarkers in the differential diagnosis of Alzheimer's disease from other cortical dementias. *J Neurol Neurosurg Psychiatry* 82**,** 240-246. doi: 10.1136/jnnp.2010.207183.

Duits, F.H., Teunissen, C.E., Bouwman, F.H., Visser, P.J., Mattsson, N., Zetterberg, H., Blennow, K., Hansson, O., Minthon, L., Andreasen, N., Marcusson, J., Wallin, A., Rikkert, M.O., Tsolaki, M., Parnetti, L., Herukka, S.K., Hampel, H., De Leon, M.J., Schroder, J., Aarsland, D., Blankenstein, M.A., Scheltens, P., and Van Der Flier, W.M. (2014). The cerebrospinal fluid "Alzheimer profile": easily said, but what does it mean? *Alzheimers Dement* 10**,** 713-723 e712. doi: 10.1016/j.jalz.2013.12.023.

Engelborghs, S., De Vreese, K., Van De Casteele, T., Vanderstichele, H., Van Everbroeck, B., Cras, P., Martin, J.J., Vanmechelen, E., and De Deyn, P.P. (2008). Diagnostic performance of a CSF-biomarker panel in autopsy-confirmed dementia. *Neurobiol Aging* 29**,** 1143-1159. doi: S0197-4580(07)00055-3 [pii];10.1016/j.neurobiolaging.2007.02.016 [doi].

Ewers, M., Mattsson, N., Minthon, L., Molinuevo, J.L., Antonell, A., Popp, J., Jessen, F., Herukka, S.K., Soininen, H., Maetzler, W., Leyhe, T., Burger, K., Taniguchi, M., Urakami, K., Lista, S., Dubois, B., Blennow, K., and Hampel, H. (2015). CSF biomarkers for the differential diagnosis of Alzheimer's disease. A large-scale international multicenter study. *Alzheimers Dement*. doi: 10.1016/j.jalz.2014.12.006.

Gabelle, A., Dumurgier, J., Vercruysse, O., Paquet, C., Bombois, S., Laplanche, J.L., Peoc'h, K., Schraen, S., Buee, L., Pasquier, F., Hugon, J., Touchon, J., and Lehmann, S. (2013). Impact of the 2008-2012 French Alzheimer Plan on the use of cerebrospinal fluid biomarkers in research memory center: the PLM Study. *J Alzheimers Dis.* 34**,** 297-305. doi: C0W1723KN16877R4 [pii];10.3233/JAD-121549 [doi].

Gabelle, A., Roche, S., Geny, C., Bennys, K., Labauge, P., Tholance, Y., Quadrio, I., Tiers, L., Gor, B., Boulanghien, J., Chaulet, C., Vighetto, A., Croisile, B., Krolak-Salmon, P., Perret-Liaudet, A., Touchon, J., and Lehmann, S. (2011). Decreased sAbetaPPbeta, Abeta38, and Abeta40 cerebrospinal fluid levels in frontotemporal dementia. *J Alzheimers Dis* 26**,** 553-563. doi: J7391P0833K432H6 [pii];10.3233/JAD-2011-110515 [doi].

Hulstaert, F., Blennow, K., Ivanoiu, A., Schoonderwaldt, H.C., Riemenschneider, M., De Deyn, P.P., Bancher, C., Cras, P., Wiltfang, J., Mehta, P.D., Iqbal, K., Pottel, H., Vanmechelen, E., and Vanderstichele, H. (1999). Improved discrimination of AD patients using beta-amyloid(1-42) and tau levels in CSF. *Neurology* 52**,** 1555-1562.

Irwin, D.J., Trojanowski, J.Q., and Grossman, M. (2013). Cerebrospinal fluid biomarkers for differentiation of frontotemporal lobar degeneration from Alzheimer's disease. *Front Aging Neurosci* 5**,** 6. doi: 10.3389/fnagi.2013.00006.

Lewczuk, P., Kornhuber, J., Vanderstichele, H., Vanmechelen, E., Esselmann, H., Bibl, M., Wolf, S., Otto, M., Reulbach, U., Kolsch, H., Jessen, F., Schroder, J., Schonknecht, P., Hampel, H., Peters, O., Weimer, E., Perneczky, R., Jahn, H., Luckhaus, C., Lamla, U., Supprian, T., Maler, J.M., and Wiltfang, J. (2008). Multiplexed quantification of dementia biomarkers in the CSF of patients with early dementias and MCI: a multicenter study. *Neurobiol.Aging* 29**,** 812-818. doi: S0197-4580(06)00469-6 [pii];10.1016/j.neurobiolaging.2006.12.010 [doi].

Maddalena, A., Papassotiropoulos, A., Muller-Tillmanns, B., Jung, H.H., Hegi, T., Nitsch, R.M., and Hock, C. (2003). Biochemical diagnosis of Alzheimer disease by measuring the cerebrospinal fluid ratio of phosphorylated tau protein to beta-amyloid peptide42. *Arch Neurol* 60**,** 1202-1206. doi: 10.1001/archneur.60.9.1202.

Mattsson, N., Zetterberg, H., Hansson, O., Andreasen, N., Parnetti, L., Jonsson, M., Herukka, S.K., Van Der Flier, W.M., Blankenstein, M.A., Ewers, M., Rich, K., Kaiser, E., Verbeek, M., Tsolaki, M., Mulugeta, E., Rosen, E., Aarsland, D., Visser, P.J., Schroder, J., Marcusson, J., De, L.M., Hampel, H., Scheltens, P., Pirttila, T., Wallin, A., Jonhagen, M.E., Minthon, L., Winblad, B., and Blennow, K. (2009). CSF biomarkers and incipient Alzheimer disease in patients with mild cognitive impairment. *JAMA* 302**,** 385-393. doi: 302/4/385 [pii];10.1001/jama.2009.1064 [doi].

Mulder, C., Verwey, N.A., Van Der Flier, W.M., Bouwman, F.H., Kok, A., Van Elk, E.J., Scheltens, P., and Blankenstein, M.A. (2010). Amyloid-beta(1-42), total tau, and phosphorylated tau as cerebrospinal fluid biomarkers for the diagnosis of Alzheimer disease. *Clin Chem* 56**,** 248-253. doi: clinchem.2009.130518 [pii];10.1373/clinchem.2009.130518 [doi].

Olsson, A., Vanderstichele, H., Andreasen, N., De Meyer, G., Wallin, A., Holmberg, B., Rosengren, L., Vanmechelen, E., and Blennow, K. (2005). Simultaneous measurement of beta-amyloid(1-42), total tau, and phosphorylated tau (Thr181) in cerebrospinal fluid by the xMAP technology. *Clin Chem* 51**,** 336-345. doi: 10.1373/clinchem.2004.039347.

Reijn, T.S., Rikkert, M.O., Van Geel, W.J., De Jong, D., and Verbeek, M.M. (2007). Diagnostic accuracy of ELISA and xMAP technology for analysis of amyloid beta(42) and tau proteins. *Clin Chem* 53**,** 859-865. doi: 10.1373/clinchem.2006.081679.

Schoonenboom, N.S., Pijnenburg, Y.A., Mulder, C., Rosso, S.M., Van Elk, E.J., Van Kamp, G.J., Van Swieten, J.C., and Scheltens, P. (2004). Amyloid beta(1-42) and phosphorylated tau in CSF as markers for early-onset Alzheimer disease. *Neurology* 62**,** 1580-1584.

Schoonenboom, N.S.M., Reesink, F.E., Verwey, N.A., Kester, M.I., Teunissen, C.E., Van De Ven, P.M., Pijnenburg, Y.a.L., Blankenstein, M.A., Rozemuller, A.J., Scheltens, P., and Van Der Flier, W.M. (2012). Cerebrospinal fluid markers for differential dementia diagnosis in a large memory clinic cohort. *Neurology* 78**,** 47-54. doi: 10.1212/WNL.0b013e31823ed0f0.

Seeburger, J.L., Holder, D.J., Combrinck, M., Joachim, C., Laterza, O., Tanen, M., Dallob, A., Chappell, D., Snyder, K., Flynn, M., Simon, A., Modur, V., Potter, W.Z., Wilcock, G., Savage, M.J., and Smith, A.D. (2015). Cerebrospinal fluid biomarkers distinguish postmortem-confirmed Alzheimer's disease from other dementias and healthy controls in the OPTIMA cohort. *J Alzheimers Dis* 44**,** 525-539. doi: 10.3233/JAD-141725.

Shea, Y.F., Chu, L.W., Zhou, L., Li, W.M., Lin, O.Y., Chan, M.N., Xu, A., Wong, R., Ho, T.Y., Liu, K.W., Ha, J., Daniel, T.W., Song, Y.Q., and Lam, K.S. (2013). Cerebrospinal fluid biomarkers of Alzheimer's disease in Chinese patients: a pilot study. *Am J Alzheimers Dis Other Demen* 28**,** 769-775. doi: 10.1177/1533317513504615.

Vanderstichele, H., De Vreese, K., Blennow, K., Andreasen, N., Sindic, C., Ivanoiu, A., Hampel, H., Burger, K., Parnetti, L., Lanari, A., Padovani, A., Diluca, M., Blaser, M., Olsson, A.O., Pottel, H., Hulstaert, F., and Vanmechelen, E. (2006). Analytical performance and clinical utility of the INNOTEST PHOSPHO-TAU181P assay for discrimination between Alzheimer's disease and dementia with Lewy bodies. *Clin Chem Lab Med* 44**,** 1472-1480. doi: 10.1515/CCLM.2006.258.

Wada-Isoe, K., Kitayama, M., Nakaso, K., and Nakashima, K. (2007). Diagnostic markers for diagnosing dementia with Lewy bodies: CSF and MIBG cardiac scintigraphy study. *J Neurol Sci* 260**,** 33-37. doi: 10.1016/j.jns.2007.03.016.

Welge, V., Fiege, O., Lewczuk, P., Mollenhauer, B., Esselmann, H., Klafki, H.W., Wolf, S., Trenkwalder, C., Otto, M., Kornhuber, J., Wiltfang, J., and Bibl, M. (2009). Combined CSF tau, p-tau181 and amyloid-beta 38/40/42 for diagnosing Alzheimer's disease. *J Neural Transm* 116**,** 203-212. doi: 10.1007/s00702-008-0177-6 [doi].

Yakushev, I., Bartenstein, P., Siessmeier, T., Hiemke, C., Scheurich, A., Lotz, J., Fellgiebel, A., and Muller, M.J. (2010). Cerebrospinal fluid tau protein levels and 18F-fluorodeoxyglucose positron emission tomography in the differential diagnosis of Alzheimer's disease. *Dement Geriatr Cogn Disord* 30**,** 245-253. doi: 10.1159/000320206.
